# Supplementary material for: Unravelling the Complexity of the +33 C>G [HBB:c.-18C>G] Variant in Beta Thalassemia
Source: Biomedicines. 2024 Jan 27;12(2):296. doi: 10.3390/biomedicines12020296 (PMC10886608; doi:10.3390/biomedicines12020296)
Supplement: Supplementary file 1 [file biomedicines-12-00296-s001.zip › biomedicines-2781609-supplementary.pdf]

Unravelling the complexity of the +33 C>G [HBB:c.-18C>G] variant in beta thalassemia

STEPHANOU Coralea[1,‡,*], PETROU Miranda[1,‡], KOUNTOURIS Petros[1], MAKARIOU Christiana[2], CHRISTOU Soteroula[2], HADJIGAVRIEL Michael[3], KLEANTHOUS Marina[1,§], PAPASAVVA THESSALIA[1,§]

[1] Molecular Genetics Thalassemia Department, The Cyprus Institute of Neurology and Genetics, Nicosia, Cyprus

[2] Thalassemia Clinic Nicosia, Archbishop Makarios III Hospital, Nicosia, Cyprus

[3] Thalassemia Clinic Limassol, Limassol General Hospital, Limassol, Cyprus

[‡] Joint first authorship

[§] Joint last authorship

[*] Correspondence should be addressed to:

Coralea Stephanou,
The Cyprus Institute of Neurology & Genetics,
6 Iroon Avenue, 2371 Ayios Dometios, Nicosia, Cyprus.
e-mail: coraleas@cing.ac.cy

Figure S1. Cypriot families with β +33 C>G [HBB:c.-18C>G].

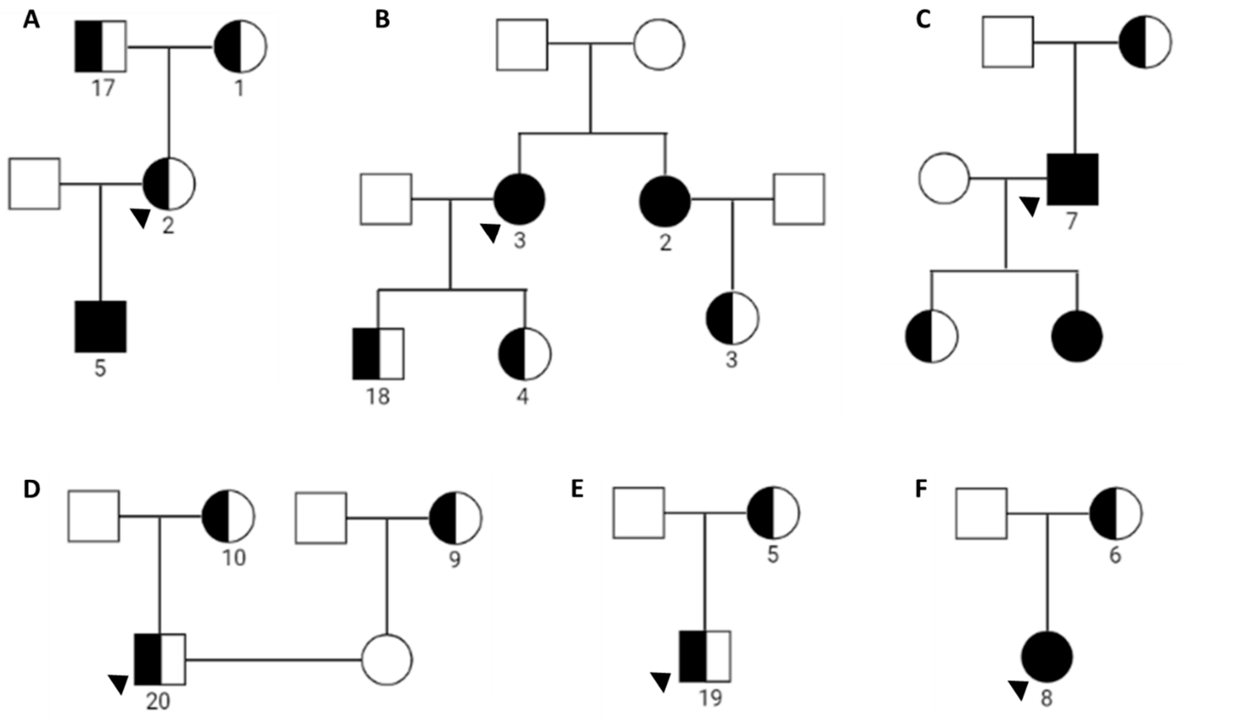

Detection of HBB:c.-18C>G in six Cypriot families (A – F) by direct sequencing. Numbers correspond to cases in Tables 3 (*HBB* heterozygotes) and 4 (*HBB* compound heterozygotes). male, female, not genotyped; , , *HBB* heterozygote; , , *HBB* compound heterozygote; proband. Pedigree structures were generated using bioRender.com.
